# Neuroinflammatory Mechanisms and Vocalization Biomarkers in the *Pink1*−/− Preclinical Model of Parkinson’s Disease

**DOI:** 10.18103/mra.v13i10.6972

**Published:** 2025-10-24

**Authors:** Sarah A. Lechner, Cynthia A. Kelm-Nelson

**Affiliations:** 1 Department of Surgery, Division of Endocrine Surgery, University of Wisconsin, Madison, Wisconsin, USA

**Keywords:** Parkinson disease, rat, biomarker, neuroinflammation, ultrasonic vocalization

## Abstract

Parkinson’s disease is a progressive neurodegenerative disorder with an unclear etiology and a long preclinical phase. The multitude of challenges of studying this in humans highlight the need for robust preclinical animal models that accurately reflect the disease’s preclinical progression. While traditional neurotoxin models fail to capture the slow, genetic nature of PD, genetic models, such as the phosphatase and tensin homolog-induced putative kinase 1 (*Pink1*−/−) knockout rat, offer a preclinical, translational approach. This review synthesizes the current understanding of the *Pink1*−/− rat model, focusing on two key aspects: neuroinflammation and vocalization deficits. The *Pink1*−/− rat presents early non-motor symptoms, including vocalization deficits, which can serve as a non-invasive, objective, and longitudinal biomarker of disease progression. This review further proposes a direct link between the model’s neuroinflammatory profile, characterized by chronic glial activation and the upregulation of inflammatory pathways, and the observed vocal dysfunctions. By bridging these two critical areas, neuroinflammation as a pathological mechanism and vocalizations as a functional readout, this review provides a framework for future investigations into the diagnosis and treatment of Parkinson’s disease and suggests that targeting specific inflammatory pathways could be a viable therapeutic strategy.

## 1. Introduction

Parkinson’s disease (PD) is the fastest-growing neurodegenerative disorder in the US^1^, with an annual incidence of 60,000 to 95,000 new diagnoses. Accelerating prevalence is due to an aging population, increased longevity, increased awareness, and unclear environmental factors^2–4^. PD places a substantial burden on the U.S. healthcare system; for example, the total annual economic burden is $51.9 billion^5^. Traditionally, the disease is characterized by the selective loss of dopaminergic neurons in the substantia nigra pars compacta, the accumulation of misfolded proteins, primarily *α*-synuclein, into Lewy bodies, as well as the ascending spread of these pathologies throughout the CNS^6–8^. While the cardinal motor symptoms, bradykinesia, rigidity, and tremor, are the most recognized features of PD in the mid-to-late stage, a wide range of debilitating non-motor symptoms, including vocalization and swallowing deficits, cognitive impairments, autonomic dysfunction, and mood disorders, often precede the limb motor deficits and are underdiagnosed yet significantly and negatively impact a patient’s quality of life^8,9^. A growing body of evidence suggests that non-motor symptoms can serve as highly sensitive and early biomarkers of PD. Of interest in this review is vocal dysfunction (dysarthria) as an early, progressive preclinical biomarker. Vocal deficits manifest preclinically and affect nearly 90% of individuals with PD; negatively impacting social interactions and quality of life. These changes include hypophonia, reduced intelligibility, and dysphonic voice characterized by rough, breathy, and monotone qualities (hypokinetic dysarthria) and are difficult to treat^10^. The complex timeline and etiology of PD remains a subject of research, with evidence pointing to a convergence of genetic predispositions with environmental factors that drive disease pathogenesis throughout a long preclinical phase.

Preclinical PD is inherently difficult to study in humans due to variable symptom manifestation, inconsistent age of onset, and environmental variability. Thus, genetic animal models offer homogeneity and superior experimental control, facilitating meaningful advancements to our understanding of PD along with the development of novel therapeutics that cannot be achieved in human studies alone^11–13^. However, a significant challenge in the field has been the lack of a single research model that fully recapitulates the progressive early onset pathology, with neurodegeneration, and both the motor and non-motor symptoms of the human disease^14^. Traditional neurotoxin-based rodent models, such as those using 6-hydroxydopamine (6-OHDA) or 1-methyl-4-phenyl-1,2,3,6-tetrahydropyridine (MPTP), induce acute and rapid dopamine neurodegeneration but often fail to model the progressive nature and genetic complexity of PD. Genetic rodent models, on the other hand, can provide a more accurate representation of the disease’s slow progression, complex biology, and can be particularly useful for investigating early, preclinical pathological events^11,15,16^. For example, mitochondrial dysfunction is a common feature of both sporadic and genetic PD^17,18^ and proteins encoded by PD-associated genes participate in molecular pathways that produce pathology indistinguishable from sporadic forms^19^. While much of the prior research has focused on dopamine-related pathophysiology and clinical time periods, there is a critical research need to better understand the mechanisms that contribute to preclinical behavioral signs. Ultimately, a genetic preclinical model advances scientific understanding that drives new devices, behavioral strategies, and drug therapies in humans.

Inherited mutations have been found to account for 4%–9% of human cases with early onset PD^20^. Yet, risk variants could explain up to 30% of the heritable risk of PD depending on prevalence^[127]^. In the preclinical phase, individuals do not exhibit clinical signs or symptoms which makes studying this phase in humans nearly impossible^[128]^. Among the current genetic models, the phosphatase and tensin homolog (PTEN)-induced putative kinase 1 [PTEN (phosphatase and tensin homologue)-induced kinase1; PARK6] knockout (*Pink1*−/−) rat has emerged as a particularly valuable research tool to study preclinical PD and associated behavioral dysfunction^12^. Loss-of-function mutations in the *PINK1* gene are a leading cause of early-onset familial PD in humans, highlighting its role in mitochondrial quality control^20,21^. Likewise, human PINK1 monogenetic variants appear similar to spontaneous forms of the disease in that they both exhibit a progressive loss of function and nigrostriatal dopamine cell loss in late-stage disease^22^. They also present with other motor and non-motor deficits, such as early-onset bulbar sensorimotor issues like speech and swallowing dysfunction, that may be linked to non-dopaminergic mechanisms^23^. Similar overlapping vagal-nerve behaviors including swallowing, chewing, and GI dysfunction with corresponding brainstem nuclei findings are also present in this model^24–29^. Research demonstrates that the *Pink1*−/− rat model develops age-dependent mitochondrial dysfunction, oxidative stress, and subsequent dopaminergic neurodegeneration, mirroring longitudinal aspects of the human disease^30–32^.

Mitochondrial dysfunction, neuroinflammation, oxidative stress, and differential modulation of the CNS immune system, plays an important role in the initial insult, spread, and severity of PD and epidemiological studies suggest that anti-inflammatory drugs may be therapeutic and reduce the incidence of PD^33,34^. For example, PD patients exhibit high levels of pro-inflammatory cytokines including TNF-α, IL-1β, and IL-6 in the brain and CSF^35^, as well as microglia activation and peripheral immune cell infiltration^36^. It is now understood that chronic activation of glial cells, particularly microglia and astrocytes, contributes significantly to neuronal damage and death. This review aims to synthesize the current understanding of the *Pink1*−/− rat model, focusing on the interplay between its specific neuroinflammatory profile and the potential of vocalization changes as a novel biomarker. By bridging these two critical areas of research, neuroinflammation as a pathological mechanism and vocalizations as a functional readout, we seek to provide a comprehensive framework for future investigations into the diagnosis and treatment of PD.

## 2. The *Pink1*−/− Rat: A Progressive Model for Preclinical Vocal Biomarkers

In humans, preclinical vocal deficits are difficult to characterize and nearly impossible to investigate due to factors like symptom manifestation, inconsistent age of disease onset, and environmental variability. And, research also shows a significant effect of sex in the incidence, clinical manifestation, and progression of PD signs, including these functional vocal changes^37–48^. Developed over a decade ago, the *Pink1*−/− rat model has been a valuable tool for studying PD and vocalizations^49^. The model closely reflects the preclinical pathology and behavioral dysfunction seen in human PD, including subtle limb motor deficits and other non-motor symptoms, described below. This wide range of observable signs makes it an ideal model for studying the full spectrum of preclinical PD pathology. While the *Pink1*−/− mouse model has also been studied^50^, the rat’s larger size, longer life span, and ability to readily train to behavioral tasks make it a better analog for humans. Additionally, the primary advantage of a genetically engineered model is the homogeneity of the sample that cannot be assessed with human post-mortem tissue and the ability to control for environmental conditions with the effect of biological sex as variable^15,16,51^. Thus, the rat provides superior experimental control and allows for meaningful contributions to our understanding of vocal communication and disease pathology. Specifically, over the last decade, the *Pink1*−/− rat has been valuable for PD-voice literature by contributing to the first characterization of vocal communication in the preclinical stage, longitudinal assessments, the development of an exercise training paradigm, all in combination with the investigation of the central nervous system pathological mechanisms.

### 2.1 RAT VOCALIZATIONS ARE ANALOGOUS TO HUMAN COMMUNICATION

Rats are social prey mammals that use ultrasonic vocalizations (USVs), occurring above the hearing range of many predators, to communicate with conspecifics. Rats generally use three classes of USVs depending on age and context. Among several call types, adult rat social USVs occur in the 50-kilohertz (kHz) range^52–55^ and are in many ways analogous and relevant to studying human vocal communication. These rat calls are affiliative and at least partially semiotic. For example, seminal literature demonstrates the rewarding and positive emotional significance of 50-kHz calls and have described how these calls are made in “appetitive” or “prosocial” situations that influence other rats’ behavior, causing them to approach^52–58^. Moreover, the production of rat USVs shares several key characteristics with human vocalization. Rat USVs are generated within the larynx on an egressive airflow, the forced flow of air through the vocal fold^59,60^, and modulated largely by the thyroarytenoid muscle^55–58^. A shared feature between USV production and human vocalization is the generation of airflow through lung pressure, which then modulates the vocal tract during exhalation with the help of intrinsic laryngeal muscles. In rats, vocal frequency changes are attributed to manipulations of this laryngeal framework. Reide and others proposed an edge-tone mechanism via control of intrinsic laryngeal muscles (via a ventral pouch) which is the foundation for vocal variation^61,62^. Recent work by Håkansson et al., however, has hypothesized that vocalizations are produced by a glottal jet impinging on the thyroid inner wall^63^. Despite these differences between human and rodent laryngeal and aerodynamic vocal anatomical geometry, there is utility in using rodents for investigating the descending CNS motor pathway, specifically in disease.

In both humans and rats, the brainstem vocal motor pathway, a highly evolutionarily conserved neural circuit, is mechanistically responsible for phonation and from a systems biology standpoint useful for studying mechanistic circuitry^59,60,64–67^. As a result, this framework is used to link alterations in muscle and central nervous system transcriptomics and proteomics to USV vocal behavioral changes in the rat^55,68^. Because of these anatomical similarities, the study of rat USVs then provides a valuable translational model for investigating vocal deficits that, addressed above, are a common sign of preclinical PD.

### 2.2 PINK1−/− RATS DEMONSTRATE PRECLINICAL VOCALIZATION CHANGES

Both male and female *Pink1*−/− rats exhibit progressive vocalization deficits compared to wildtype control rats^28,69,70^ and the age of onset and how the vocalization parameters change with age significantly differs between sexes^69^. The first data published by Grant 2015 et al., showed that male *Pink1*−/− rats exhibit vocalization changes to intensity (loudness), peak frequency, and bandwidth and that these vocal deficits persist and worsen from 2 months to 8 months of age. Since then, these data have been replicated in additional studies and experiments. At 10 months of age, male *Pink1*−/− rats were tested in combination with respiratory measures and the data show that respiration is increasingly variable; variability in motor behaviors (a phenotype of this model) is increased in *Pink1*−/− rats^71^. Lesser data exist in female *Pink1*−/− rats; however, Marquis 2018 et al. showed that from the onset of testing, these rats have reduced intensity but no significant differences in other acoustic and non-acoustic measures over time. In an ethological context, data suggest that wildtype female rats show decreased motivation to approach *Pink1*−/− vocalization stimuli, suggesting that these deficits impair the social purpose in this model and with our other data that they are measurably different. Male responses to female *Pink1*−/− rats have not yet been studied^72^.

### 2.3 PINK1−/− RATS CAN BE VOCAL TRAINED AS A PRECLINICAL MODEL OF THERAPY

Once diagnosed with a voice disorder, humans are often offered behavioral intervention. LSVT LOUD^®^ is the gold-standard, high-intensity vocal-exercise speech training in humans that focuses on improving vocal loudness by adjusting sensorimotor perception via retraining processes including sensory recalibration and training in self-cueing and attention^73^. During training, 16 individual hour-long sessions are delivered over four weeks by a certified therapist with additional daily tasks for the patient to complete. The effects of training-include increases in loudness (amplitude of respiratory-laryngeal movement) that can be maintained for 12–24 months^74,75^. LSVT LOUD^®^ also has some effects on pitch variability, articulatory precision, and intelligibility. Importantly, LSVT LOUD^®^, when successful, increases communication effectiveness, vocal ability, and participation in social conversation^76,77^. We have mimicked this regime in the *Pink1*−/− rats which is especially relevant because it allows for simultaneous study of behavioral benefits with CNS mechanisms and alterations (see below). This validated behavioral paradigm is systematic, repeated, and uses controlled activation of groups of muscles for sequences of goal-directed actions.

Yang 2015 et al. first showed that *Pink1*−/− rats can be trained using a vocal exercise paradigm where rats are water restricted and trained to perform a socially motivated increase in vocalizations. Then, vocal parameters, such as loudness, are recorded and analyzed by researchers. The data show that there is a significant increase in loudness (intensity) and bandwidth (frequency range) with 2 weeks of training. However, despite continued daily training these effects do not last beyond 2 months and then decline. Thus, like in humans the responses can be heterogenous and as the disease progresses non-responsive to sustained therapeutic levels of vocal training. More recently, *Pink1*−/− rats that were both vocal and tongue trained (a form of swallowing exercise) produced greater tongue forces, faster tongue contraction, as well as higher-intensity vocalization following exercise between 2 and 4 months of age. However, at 6 months of age, vocal exercise resulted in increased call complexity but did not change intensity. Then at 10 months, vocal exercise no longer influenced vocalization complexity. This suggests that there is potential to change behavioral phenotypes in the very early preclinical period but that adaptation or combination therapies in the later stages may be necessary.

### 2.4 PINK1−/− RATS HAVE PRECLINICAL BRAINSTEM PATHOLOGY THAT CORRELATES TO BEHAVIORAL DEFICITS

Much like humans, USV deficits in *Pink1*−/− rats appear independent of severe nigrostriatal dopamine loss and they do not respond to L-dopa or methylphenidate (noradrenergic) treatment^78,79^. Yet, a limitation of this *Pink1*−/− rat model, in addition to increased variability in motor performances, has been the inconsistent reporting of nigral dopamine depletion^[74]^. The first reports suggested male rats had a 50% reduction in dopamine cells by 8 months of age but this has not been replicated since^[43,74]^. However, those first studies also investigated vocal parameters and limb motor movements in correlation with locus coeruleus tyrosine hydroxylase immunohistochemical cell counts and optical density measurements. *Pink1*−/− rats have a significant reduction in the number of noradrenergic cells in the locus coeruleus^28^. This research also showed the presence of aggregated α-synuclein in locus coeruleus, nucleus ambiguus, substantia nigra, and the periaqueductal gray^[63,73]^. And, [18F]NS12137 PET imaging has demonstrated a reduction in norepinephrine transporter binding in other brain regions including the thalamus and prelimbic area^[75]^. Other research showed that along with vocal exercise intervention in *Pink1*−/− rats, there was correlational mesolimbic gene changes; for example, there is increased GABAergic gene expression within the ventral tegmental area of vocal-exercised *Pink1*−/− male rats directly suggesting that vocal training increases GABA mRNA expression^70^. More recent studies have used other molecular assays including high-performance liquid chromatography (HPLC) to study the catecholamine neurotransmitter microenvironment of the CNS immediately after behavioral data collection which found a reduction in serotonin in the brainstem of *Pink1*−/− rats^80^. Together, the catecholamine early pathology suggests a model which is more consistent with early Braak PD staging^6,7^.

### 2.5 GENE TRANSCRIPTOME WORK CAN LINK BRAINSTEM BIOLOGICAL NETWORKS TO VOCALIZATION DYSFUNCTION

Finally, recent work shows that *Pink1*−/− vocal deficits are concurrent with gene transcriptional changes in head/neck muscles and lower brainstem regions (*e.g.* decreased norepinephrine, α-synuclein accumulation, increased inflammation) and brainstem cell loss^25,28,29,69,72,81,82^. Current research used RNA sequencing (RNA-seq) to evaluate differences in gene expression and use statistical modeling to incorporate vocal behavioral datasets. For example, within the periaqueductal gray in male and female *Pink1*−/− rats compared to wildtype controls, we showed specific gene expression modules correlated with vocalization, particularly in female rats.^83^ Importantly, the identified differentially expressed genes in both sexes were found to align with data from human PD patients, highlighting the translational value of the rat model for studying the disease. This also sets the stage for the hypothesis that there are observable predictors of prodromal PD behavior. Since, RNA-seq has been used by Lechner et al. to evaluate blood, muscle, and brain transcriptomic changes^83–86^. These findings led to the hypothesis that neuroinflammation is present in the *Pink1*−/− model, contributes to prodromal-like behaviors, and may serve as a therapeutic target. Supporting this, our transcriptome-based drug repurposing database identified mostly anti-inflammatory and antioxidant compounds, further corroborating the hypothesis.

## 3. The Role of Neuroinflammation in Parkinson’s Disease Pathogenesis

While the hallmark neuropathology of PD has been identified as the loss of dopaminergic neurons in the substantia nigra, seminal literature has shown that preclinical PD is evident in lower brainstem regions prior to cortico-basal ganglia involvement and overtime the disease spreads rostrally^6,7^. This pathology is widespread and includes the disruption of neurotransmitters and accumulation of abnormal α-synuclein protein aggregation which has been observed at multiple levels in both the central and peripheral nervous system^87,88^. The mechanisms preceding aggregate formation and nigral neuronal cell loss are largely unknown but neuroinflammation is known to play a significant role in the neurodegeneration in PD, glia activation and upregulation of pro-inflammatory cytokines in the brainstem.

Within the CNS, the two main drivers of early neuroinflammation, brain-resident microglia and astrocytes^89,90^, undergo transcriptional reprogramming in preclinical disease^91–93^. Over time, the new pathology associated with PD activates further microglia responses in a cyclical pattern; this has been reported within the substantia nigra of PD patients^94^. In patients with early PD diagnoses, the midbrain is densely populated with activated microglia^95^, and, in late PD, postmortem human brain tissue demonstrates elevated levels of IL-2, IL-6, and TNF-α as well as IL-1β in the substantia nigra^96,97^. Moreover, single-cell RNA sequencing of nigral tissue also demonstrates neurodegenerative contributions of microglia as well as astrocytes^98,99^.

This is also replicated in animal models where injections of PD-like synuclein microfibers results in microgliosis^100^. In the 6-OHDA rat lesion model of PD, reactive microgliosis precedes the onset of dopamine cell death^101^. Similar findings in genetically modified mouse models expressing human wildtype or mutant α-synuclein demonstrate increased microgliosis and proinflammatory cytokine levels from astrocytes in the brainstem. Other Pink1/Parkin modeling has shown increased ROS and interleukins in the CSF in a stress/insomnia model suggesting that this pathway, while not unique to PD, has direct implications on mitochondrial autophagy in the CNS^102^. Ultimately, abnormalities within the CNS lead to inflammatory responses which are likely to exacerbate early PD leading to disease progression over time.

### 3.1 NF-ΚB AND MAPK SIGNALING ARE UPREGULATED IN PD

The transcription factor NF-κB (nuclear factor κ-light-chain-enhancer of activated B cells) and MAPK (mitogen-activated protein kinase) pathways have both been implicated in the neuropathogenesis of PD for their roles in innate immune responses, cell differentiation, stress response, autophagy, and neuronal death^103^. A study by Saijo et al. suggested that the NF-κB pathway contributes to the enhancement of neurotoxic inflammatory responses resulting in increased toxicity and subsequent dopaminergic cell death^104^. Together, in response to insult or PD disease properties, activation of these two pathways triggers the production of cytotoxic moieties and enzymes including reactive oxygen species (ROS), nitric oxide synthase (NOS), and pro-inflammatory cytokines, all of which contribute to neuronal dysfunction and ultimately cell death. Evidence from both human studies and animal models suggest that these inflammatory responses are cofactors in PD pathology and PD disease severity^105^. For example, both NF-κB and MAPK have been linked to α-synuclein pathology in the early stages of PD and are hypothesized to form this positive feedback loop^6,7^. In addition, MAPK inhibitors have demonstrated promising neuroprotective properties by restoring nonmotor symptoms of PD such as cognitive impairments and reduced α-synuclein aggregation and neuronal cell death^106^. Mitochondrial dysfunction also occurs in other neurodegenerative disorders including amyotrophic lateral sclerosis, Alzheimer’s, Huntington’s as well as eye diseases such as age-related macular degeneration and glaucoma with a common link being PINK1/PARKIN-mediated neurodegeneration and neuroinflammation.

### 3.2 LOSS OF PINK1 EXACERBATES NEUROINFLAMMATION

As discussed earlier, mutations in *PINK1* cause a heritable form of early-onset PD. In normal functions, PINK1 accumulates damaged mitochondria acting as a flag for proteolysis; it regulates mitochondrial quality control via its ability to promote mitophagy of depolarized mitochondria^107^. PINK1 acts in coordination with PARKIN, an E3 ubiquitin ligase, to label damaged mitochondria for degradation via fusion with lysosomes, forming autophagosomes^108^. Research shows that impaired mitochondrial function contributes to the formation of protein aggregates, and accumulated aggregates damage mitochondria, forming a self-perpetuating, toxic cycle that ultimately leads to cell death^109^. The PINK1/Parkin-mediated mitophagy contributes to PD^110^ and, again, increased inflammation is hypothesized to play a key role in the onset of the disorder by specifically leading to increased ROS, oxidative stress, and abnormal mitochondrial functioning and these byproducts of cellular metabolism cause significant cellular damage^111,112^. For example, *PINK1*−/− cell cultures of mixed astrocytes and microglia have increased iNOS, NO, TNF-α, and reduced IL-1β expression^113^. The loss of PINK1 in astrocyte cell culture increases ROS and induces NF-κB signaling^97^. Additionally, rodent studies in *Pink1*−/− mice have shown that loss of Pink1 increases the expression of pro-inflammatory cytokines such as TNF-α, IL-1β, and IL-6 compared to wildtype control mice^114^. Another study also showed that *Pink1*−/− mice also had increased levels of IL-12 and IL-10 in addition to IL-1β in the striatum. It has been hypothesized that the early loss of Pink1 causes a severe disruption in expression of genes that regulate innate immune systems leading to inflammation-induced cell death with time^115^.

### 3.3 NEUROINFLAMMATION MAY BE THE LINK TO PD BEHAVIOR

The link between neuroinflammation and behavior deficits in PD is largely understudied. However, some studies have shown that preclinical inflammation may modulate behavior^116–118^. In humans, preclinical PD biomarkers of neuroinflammation^119^ are associated with non-motor behavioral changes including rapid eye movement sleep behavior disorder (RBD) and cognitive function^9,120^. Meanwhile, rodent models are an invaluable tool that allow for the sequential study of disease onset and progression via a plethora of behavioral outcomes as well as specific brain biology within the same individual. For example, mice deficient for the c-Rel/NF-κB transcription factor develop olfactory and gastrointestinal dysfunction and demonstrate preclinical Braak staging brain pathology^121^. These data demonstrate that dysregulation in the NF-κB pathway may cause nonmotor PD deficits. Additionally, in rats, rotenone-induced PD causes increased ROS production which activates the MAPK signaling pathway leading to stress-induced dopaminergic cell death and impaired movement in behavioral assays (*i.e.* open field, rotarod)^122^. AAV overexpression of α-synuclein leads to activated microglia with preclinical behavioral deficits further demonstrating a PD: inflammation link^123^. However, due to the systemic nature of immune progression in these models, it is particularly challenging to determine whether changes began in the periphery and progressed to the CNS or vice versa. And, to-date, no studies have examined brainstem inflammation in the context of preclinical vocal dysfunction.

The release of pro-inflammatory cytokines and chemokines, and their contribution to neurodegeneration is well studied. However, the use of the *Pink1*−/− rat model allows us to research the complex, bidirectional relationship between mitochondrial dysfunction and the inflammatory cascade in a preclinical animal model. We propose that targeting these specific inflammatory pathways could be a viable therapeutic strategy for vocal deficits, specifically in the preclinical time period.

## 4. Linking Neuroinflammation to Vocal Dysfunction in the *Pink1*−/− Rat Model

We hypothesize a direct link between the neuroinflammatory processes in the *Pink1*−/− rat and the observed changes in vocalization behavior and acoustic parameters. The chronic inflammatory environment induced by the absence of Pink1, particularly in vocal-motor pathways, likely contributes to the neural circuit dysfunction that manifests as altered vocalizations.

### 4.1 PINK1−/− RATS HAVE UPREGULATED INFLAMMATION BIOINFORMATICALLY CORRELATED TO VOCALIZATION

A bioinformatic approach to analyzing gene expression pathways of mitochondria dysfunction, inflammation markers, and biological pathways associated with inflammation correlate to vocalization changes and vocal fold biology in *Pink1*−/− rats. Additionally, we have consistently demonstrated upregulated inflammation pathways in brain, blood, and muscle tissue, including TNF-α, NF-κB signaling and MAPK signaling in both male and female *Pink1*−/− rats.

Peripheral changes in vocal fold musculature in *Pink1*−/− rats include increased centralized nuclei^25,124^. Centralized nuclei in the vocal fold has been used to describe the nuclear positioning in muscle fibers and in adult PD, may be reflective of muscle regeneration and rearrangement and an ongoing biomarker for muscle health^125,126^. While this measure isn’t a direct result of inflammation, it is an indicator of muscle involvement. Additionally, within the male *Pink1*−/− vocal fold, there were 134 annotated differentially expressed genes and observed enrichment in the biological pathways including Parkinson’s disease (*Casp7*, *Pink1*); Parkin-Ubiquitin proteasome degradation (*Psmd12*, *Psmd7*); MAPK signaling (*Casp7*, *Ppm1b*, *Ppp3r1*); and inflammatory TNF-α, NF-κB Signaling (*Casp7*, *Psmd12*, *Psmd7*, *Cdc34*, *Bcl7a*, *Peg3*)^86^. *Casp7* was upregulated in the majority of these pathways and plays a significant role in the modulation of the cell cycle, specifically, apoptosis. In female *Pink1*−/− rats, significant upregulated pathways included fatty acid oxidation and muscle contraction, synaptic transmission, and neuromuscular processes^84^. We use Weighted Gene Co-expression Network Analysis (WGCNA) to construct gene co-expression modules to identify statistical correlations between biological gene networks with vocal behavioral data. A 2020 gene expression study in the PAG of both male and female *Pink1*−/− rats also linked co-expression modules to vocalization dysfunction^83^. *Pink1* itself was the central node with the highest number of interactions with other genes including solute carriers, glutamate metabotropic receptors, and genes associated with protein localization. In females several different gene modules (regulation of membrane depolarization (GO:0003254); include monoamine transport (GO:0015844); generation of neurons (GO:0048699); dopamine transmembrane transporter activity (GO:0005329); nervous system development (GO:0007399), axon guidance (GO:0007411); mRNA splicing (GO:00003898); and neuropeptide hormone activity (GO:0005184) were significantly correlated with vocalization traits including duration, intensity, bandwidth, and peak frequency^83^. Using vocal fold gene data with drug repurposing databases, we found several drug treatment options including cetuximab, fluoxetine, and resveratrol are hypothesized to reverse observed genetic dysregulation; anti-oxidant, anti-inflammatory compounds also target the neuroinflammatory processes in the model^83^.

Increased levels of TNF-α is mitogenic to astrocytes and is associated with the overexpression of astrocytic glial fibrillary acidic protein (GFAP). Using our brain and vocal fold sequencing data sets, we suspect that increased production of TNF-α may upregulate Gfap through the MAPK signaling pathway^127,128^. We hypothesize that biomarkers of astrocyte inflammation (via increases in Gfap), are a contributing factor in the pathogenesis of early-onset disease and may contribute to early, nonmotor deficits including dysphonia, dysarthria, and cognition deficits^129^. For example, PD inflammation processes within the vocal folds may lead to supraglottal hyperadduction, which can change the structure and function of the speech musculature^130^.

### 4.2 YOUNG PINK1−/− RATS SHOW INFLAMMATION BEFORE BEHAVIORAL SIGNS

In the earliest timepoint examined, 3 months of age, we further show that inflammation and interferon signaling upregulation in the whole blood is present during early, preclinical PD^131^. Specifically, this includes those related to type I interferon signaling (*Ifit1bl*; interferon-induced protein with tetratricopeptide repeats 1B-like), type II interferon signaling (*Nos2*; nitric oxide synthase 2), and apoptosis (*Dedd2*; death effector domain containing 2). The lack of Pink1 may cause an early disruption in interferon signaling that leads to downstream overproduction of proinflammatory cytokines (TNF-α, IL-1β, IL-6) and worsen over time, leading to neuronal cell death.

### 4.3 TREATMENT DEVELOPMENT SHOULD FOCUS ON INFLAMMATION AND NEUROPROTECTION IN THE PRECLINICAL RAT

Genes and pathways identified with transcriptome studies are useful for evaluating the mechanisms of peripheral dysfunction including within the vocal fold muscle and have potential to be used as experimental biomarkers for the development of vocal dysfunction treatments. Alternative therapeutic interventions aiming to modulate PINK1/Parkin signaling might have the potential to treat preclinical PD. For example, AAV vectors and gene augmentation has been shown to reduce neuropathology^132^, induction of overexpression of Parkin is neuroprotective^133^, and there may be benefits of antioxidant supplementation-based strategies to combat Pink1-mediated gliosis^134^. Previous RNA sequencing and bioinformatics analyses revealed that both male and female *Pink1*−/− rats exhibit significantly upregulated neuroinflammatory gene networks, strongly influenced by histone deacetylases, in the brainstem. Epigenetic alterations, specifically histone modifications, are implicated in human PD and PD animal models also show that HDAC6 plays a role in the pathogenesis of PD. Therefore, histone deacetylase inhibitors (HDACi), such as Vorinostat (suberoylanilide hydroxamic acid; SAHA), may be promising as a therapeutic. SAHA is an epigenetic-modifying class I/ class II HDACi and specific HDAC6 inhibitor that is neuroprotective, reduces CNS inflammatory responses, and induces epigenetic reprogramming. These therapeutic strategies merit further investigation in the *Pink1*−/− rat model.

## 5. Conclusions

Overall, preclinical animal models are necessary to study and evaluate inflammatory mechanisms, therapeutic treatments, safety, and efficacy to move the study of human disease forward. Genetic rodent models of PD, specifically the *Pink1*−/− rat, have been shown to be useful tools to study Parkinsonian genes and inflammatory networks, preclinical behavioral and functional vocalization changes to identify potential druggable targets for preclinical pharmacological treatments. Progressive vocalization changes in the *Pink1*−/− rat provide noninvasive, objective measures that can be correlated to neuroinflammatory damage and neurodegeneration. Moreover, the ability to collect large amounts of USV data from rats is ideal because it is non-invasive, objective with newer machine-learning based analyses, and can be used longitudinally to track disease progression using the *Pink1*−/− model. Repeated weekly, monthly, or bimonthly testing is feasible without negative habituation to the task, and as a progressive preclinical model, very useful to correlate behavioral changes to progressive CNS changes in the same subject. Importantly, repeated subjects’ statistics allow for rigor and reproducibility in these preclinical studies. Moreover, vocal data collection can generate very large datasets, which are ideal for statistical analysis with AI machine learning applications to find subtle patterns in combination with blood, muscle, and brain inflammatory transcriptomics. Ultimately, these data and this experimental framework support the view that neuroinflammation is an early driver of disease, and targeting specific inflammatory pathways may be a promising therapeutic strategy.
